# Mater-Bi-Based Biocomposites Reinforced with Lemongrass: A Comparison Between Leaf- and Culm-Derived Particles

**DOI:** 10.3390/polym17212909

**Published:** 2025-10-30

**Authors:** Manuela Ceraulo, Luigi Botta, Carmelo Sanfilippo, Sanjay Mavinkere Rangappa, Suchart Siengchin, Vincenzo Fiore

**Affiliations:** 1 Department of Engineering, University of Palermo, Viale Delle Scienze, Edificio 6, 90128 Palermo, Italy; manuela.ceraulo@unipa.it (M.C.); luigi.botta@unipa.it (L.B.); carmelo.sanfilippo01@unipa.it (C.S.); 2 Department of Materials and Production Engineering, The Sirindhorn International Thai-German Graduate School of Engineering (TGGS), King Mongkut’s University of Technology North Bangkok (KMUTNB), 1518 Pracharat 1, Wongsawang Road, Bangsue, Bangkok 10800, Thailand; mavinkere.r.s@op.kmutnb.ac.th (S.M.R.); suchart.s@tggs.kmutnb.ac.th (S.S.)

**Keywords:** biocomposites, biodegradable polymers, thermoplastic starch, Mater-Bi, lemongrass particles

## Abstract

In this study, aiming to develop novel biocomposites that offer competitive properties while retaining their renewable and biodegradable characteristics, a biodegradable polymer matrix (Mater-Bi^®^ HF51L2) was reinforced with natural particles extracted from the culm and leaf of Cymbopogon flexuosus (lemongrass). Particles (<500 µm) were incorporated at 10 and 20 wt.% via twin-screw extrusion followed by compression moulding. Morphological analysis via SEM revealed distinct structural differences between culm- and leaf-derived particles, with the latter exhibiting smoother surfaces, higher density, and better dispersion in the matrix, resulting in lower void content. Quasi-static mechanical tests showed increased stiffness with filler content, particularly for leaf-based composites. This material, at 20 wt.% filler loadings, enhanced the tensile and flexural moduli of the neat Mater-Bi approximately three and two times, respectively, a result attributed to enhanced interfacial adhesion. Rheological measurements (rotational and capillary) indicated significant increases in complex viscosity, particularly for leaf-filled systems, confirming restricted polymer chain mobility and good matrix–filler interaction. Dynamic mechanical thermal tests (DMTA) results showed an increased storage modulus and a shift in glass transition temperature (T_g_) for all biocomposites in comparison to Mater-Bi matrix. Specifically, the neat matrix had a T_g_ of −28 °C, which increased to −24 °C and −18 °C for the 20 wt.% culm-reinforced and leaf-reinforced biocomposites, respectively. Overall, the leaf-derived particles demonstrated superior reinforcing potential, effectively improving the mechanical, rheological, and thermal properties of Mater-Bi-based biocomposites.

## 1. Introduction

The rise in environmental consciousness in recent decades has been a driving force behind the use of eco-friendly and more sustainable composite materials, commonly known as biocomposites [1,2,3,4]. While the current generation of biocomposites successfully substitutes petroleum-based polymers, a significant challenge remains: achieving truly fully bio-based systems where both the composite matrix and the reinforcing modifier belong exclusively to the family of sustainable and renewable constituents. Many current ‘bio-plastics’ are only partially derived from biomass [5,6], and many high-performance composite additives used to improve the matrix–reinforcement interface, still rely on non-renewable sources, thereby compromising the material’s overall sustainability profile [7]. The next frontier in sustainable material design, therefore, must focus on maximizing the renewable carbon content of the entire composite structure. This necessitates the rigorous investigation of novel, non-food-competitive lignocellulosic biomass to serve as effective modifiers, paired with existing, commercially scalable bio-matrices. Addressing this gap requires evidence that maximizing renewability does not compromise material performance. In such a context, several researchers focused their attention on the use of biobased and/or biodegradable polymers to fully or partially replace conventional plastics derived from oil [8,9,10] as well of natural fibers or particles as alternative reinforcement of synthetic fibers [11,12]. Specifically, several plants have been investigated as possible source for natural reinforcement in recent years [13,14,15]. Among them, lemongrass plant (*Cymbopogon genus*) seems to be a promising option. This clumped and tall grass, also known as barbed wire grass, silky heads, Cochin grass, Malabar grass, oily heads, citronella grass and fever grass [16,17], can reach heights of 1.8 m and widths of 1.2 m. It has a small rhizome, and the leaves are 1.3–2.5 cm wide and 1 m long. It belongs to the *Poaceae* family and comprises more than 55 species. The most popular are *Cymbopogon flexuosus* and *Cymbopogon citratus*, because an essential oil widely used in food preservation and perfumery is extracted from them [18]. For this scope, they are cultivated worldwide [19,20]. Moreover, lemongrass plant is used in pharmacology as well as in therapeutic applications [21,22], for the production of biogas [23] and silica [24], for paper-making [25] and for heavy metals biosorption [26]. Specifically, an our recent work has been dedicated to the comparison of natural fibers extracted from the leaf and the culm of lemongrass plant (*Cymbopogon flexuosus*) collected from local agricultural land in the area of Bangkok (Thailand). It was basically shown that, in spite of similar chemical composition, leaf fibers evidence better tensile properties than culm ones [27]. This finding was attributable to the larger amount of absorbed water and ash content shown by culm fibers in addition to a more compact structure of leaf fibers, also having higher density.

Despite a wide literature is available on natural fiber reinforced biocomposites, only a few studies to date have focused on using of fibers or particles from the lemongrass plant as reinforcement for synthetic or biopolymers [28,29,30,31,32,33]. In particular, lemongrass particles were used very recently, both independently and in combination with poplar wood particles, to manufacture particle board bonded with urea-formaldehyde resin [34].

In this study, Mater-Bi^®^ was selected as the polymer matrix. This commercial biodegradable formulation is based on blends of aliphatic–aromatic polyesters and thermoplastic starch, designed to combine biodegradability with good mechanical strength and processability. The use of Mater-Bi^®^ is particularly attractive for sustainable packaging applications, as it ensures a low environmental impact and compatibility with existing industrial processing technologies. Moreover, its partial polysaccharidic nature (due to starch content) could provide good interaction potential with natural fillers, improving dispersion and interfacial adhesion within the composite structure [35,36].

To the best of our knowledge, despite other natural fillers and fibers like grape pomace [37,38], brewers’ spent grain [39,40,41], alfa fibers [42], tomato plant [43], natural keratin, and coconut fibers [44] having already been used to evaluate their effect on the performance and sustainability of Mater-Bi based biocomposites. Additionally, systems like starch-bamboo [45], banana starch–potato peel powder [46], apricot and walnut shells [47] have also been explored to develop biocomposites with improved properties while retaining their renewable and biodegradable characteristics. However, no effort has yet been made to assess the impact of lemongrass particles.

Starting from this premise, this research aims to assess for the first time how the presence of lemongrass particles from both the culm and leaf parts modifies the properties of neat Mater-Bi, with the ultimate goal of obtaining a reliable product competitive with analogous, already investigated natural systems. To this scope, particles (<500 µm) were added at 10 and 20 wt.% via twin-screw extrusion followed by compression molding to Mater-Bi^®^ HF51L2, and the produced biocomposites were investigated form a morphological, thermal, rheological and mechanical point of view.

## 2. Experimental

### 2.1. Materials and Methods

The biodegradable polymer used as the matrix for bio-composites was thermoplastic starch commercial grade Mater-Bi^®^ HF51L2, purchased by Novamont SPA (Novara, Italy). This material has a melt flow index (190 °C/2.16 kg) of 4.5 g/10 min, a melting temperature of 150 °C, and a density of 1.21 g/cm^3^. First, fresh culms and leaves were dried at room temperature for 24 h. After drying, they were ground using a grinding machine. The resulting lemongrass particles were then sieved to obtain a fraction with a maximum size of 500 µm.

Before the composites were prepared, both the Mater-Bi matrix and the lemongrass particles were dried under vacuum at 60 °C for 12 h. This step was crucial to protect the polymeric matrix from hydrolytic scission phenomena during processing.

For both lemongrass particles, two biocomposites (containing 10 and 20 wt.% of lemongrass) were manufactured via extrusion and compression molding. Biocomposites particles were initially prepared by using a laboratory modular co-rotating twin screw extruder (OMC Italy, D = 19 mm, L/D = 35). For comparison, the neat Mater-Bi matrix was processed under the same conditions. The thermal profile adopted was 120–130–140–150–160–170–170 °C, and the rotational speed was set to 180 rpm. The molten material obtained from the extruder die was cooled on line in a water bath, pelletized, and then used for the subsequent manufacturing step.

Afterwards, the pellets obtained from extrusion were dried under vacuum at 60 °C for 12 h. Finally, samples for mechanical, rheological and morphological characterizations were prepared through compression molding (T = 160 °C, P = 27 bar, time = 3 min) using a Laboratory press by Carver (Wabash, IN, USA).

**Figure 1:**
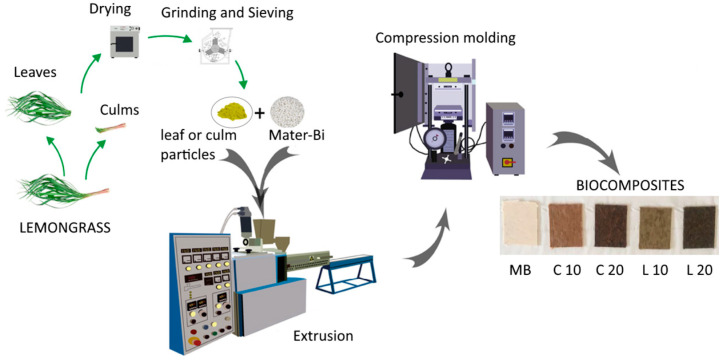
Schematic diagram for the manufacturing stages of biocomposites.

Biocomposites will be identified by codes indicating their composition: L 10, L 20, C 10, and C 20. The letter (L for leaves, C for culms) denotes the lemongrass part used, and the number (10 or 20) indicates the filler weight content. The identifying code MB refers to the neat polymeric matrix. Figure 1 illustrates the schematic diagram of each stage of the manufacturing process.

### 2.2. Morphological Analysis

The morphology of lemongrass particles and composites were observed by using Scanning Electron Microscope (SEM) model Quanta 200F by FEI (Hillsboro, OR, USA), operating at 10 kV. SEM micrographs of composites were carried out on specimens fractured in liquid nitrogen and gold-sputtered to avoid electrostatic charging under the electron beam.

### 2.3. Density and Void Content Measurements

The experimental density of each investigated composite (*ρ_ce_*) was measured by using a helium pycnometer model Pycnomatic ATC by Thermo Fisher Scientific (Waltham, MA, USA) and an analytical balance model AX 224 by Sartorius (Gottingen, Germany). For each measured sample, 10 tests were carried out and average values were recorded. All the measured standard deviations were lower than 0.01 g/cm^3^.

By applying the mixture rule the theoretical density of the composites (*ρ_ct_*) was also calculated as follows:

(1) $$\rho_{ct}=\;\frac{1}{\frac{W_{l}}{\rho_{l}}+\frac{W_{m}}{\rho_{m}}}$$

where *ρ_l_* is the experimental density of lemongrass reinforcement, i.e., 1.139 g/cm^3^ and 1.019 g/cm^3^ for leaf and culm particles, respectively [27]. On the other hand, Mater-Bi matrix showed an average experimental density (*ρ_m_*) equal to 1.259 g/cm^3^. *W_f_* and *W_m_* represent the weight content of Mater-Bi and lemongrass particles in each investigated composite, respectively.

The voids volume fraction (*V_V_*) was determined by comparing the experimental and theoretical density values, in accordance with ASTM D2734 standard, by using the following equation:

(2) $$V_{V}=\;\frac{\rho_{ct}-\rho_{ce}}{\rho_{ct}}$$

### 2.4. Rheological Characterization

The dynamic rheological behavior of both the neat matrix and its composites was analyzed using a ARES G2 rotational rheometer by TA Instruments (New Castle, DE, USA). Frequency sweep tests were conducted using the parallel-plates configuration with a 25 mm diameter and a 1.5 mm gap, over an angular frequency range of 10^−1^ to 10^2^ rad/s. A constant strain amplitude of 5% was maintained, a value determined to be within the linear viscoelastic region through preliminary strain sweep tests.

A capillary Rheologic 1000 viscometer by Ceast-Instron (Pianezza, Italy) was employed for shear flow rheological characterization. These tests were performed using a capillary die with a diameter (D) of 1 mm and a length-to-diameter ratio (L/D) of 40.

### 2.5. Mechanical Characterization

Quasi-static tensile and flexural tests were performed according to ASTM D638 and ASTM D790 international standard by using a Universal Testing Machine (U.T.M.) model Z005 by Zwick-Roell (Ulm, Germany), equipped with a load cell of 200 N. Five dumbbell samples for each composite were tested in tensile configuration by setting the crosshead speed equal to 5 mm/min for elongation percentage values up to 8% and then equal to 50 mm/min. On the other hand, flexural tests were carried out on five prismatic samples (3 mm × 12.7 mm × 60 mm) for each composite by setting the span length and the crosshead speed equal to 48 mm and 1.28 mm/min, respectively.

The dynamic properties of composites were evaluated through dynamic mechanical thermal tests (DMTA), in accordance with ASTM D4065 standard. This characterization was performed under tensile configuration at constant frequency of 1 Hz using a dynamic mechanical analyzer model DMA+50 by Metravib (Limonest, France). Three prismatic samples (0.5 mm × 6 mm × 25 mm) for each investigated composite were tested from −50 °C to 20 °C at a heating rate of 5 °C/min.

### 2.6. Calorimetric Analysis

The thermal properties of the composites were studied using a differential scanning calorimeter model DSC131 Evo by Seteram (Hillsborough Township, NJ, USA). Specimens, approximately 8 mg in weight and obtained from tensile samples, were sealed in aluminum pans. The experiments were performed under a nitrogen atmosphere with a heating cycle from 25 °C to 200 °C at a rate of 10 °C/min, followed by a cooling cycle at the same rate from 200 °C to 25 °C.

## 3. Results

### 3.1. Morphological Features

**Figure 2:**
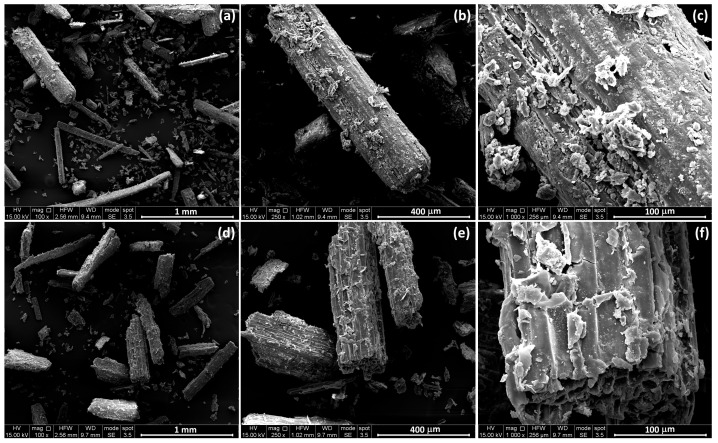
SEM micrographs of lemongrass particles at different magnifications: (**a**–**c**) culm and (**d**–**f**) leaf-derived particles.

Figure 2 shows the SEM micrographs at different magnifications of lemongrass particles obtained by grinding and sieving, respectively, culm (Figure 2a–c) and leaf (Figure 2d–f) of the plant.

In our previous work [27], the morphology of fibers obtained from lemongrass culm and leaf has been analyzed, revealing that the two fiber types exhibited distinct structures, including differences in the size, shape and arrangement of their cells, as well as the characteristics of their lumens. Specifically, culm fibers showed larger cells with two central spherical lumens, whereas leaf fibers showed smaller cells with narrower and occasionally elongated lumens.

Regarding particle dimensions, it can be observed that both culm- and leaf-derived particles appear elongated. Culm particles have an average length of 537 µm and an average diameter of 86 µm with an average aspect ratio (L/D) of 6.9, whereas leaf particles exhibit an average length of 480 µm and an average diameter of 115 µm with an average aspect ratio (L/D) of 4.5. Furthermore, impurities are present on the surfaces of both particle types, but they are more pronounced in the particles obtained from the culm. Additionally, the particles derived from the leaf display a smoother surface.

**Figure 3:**
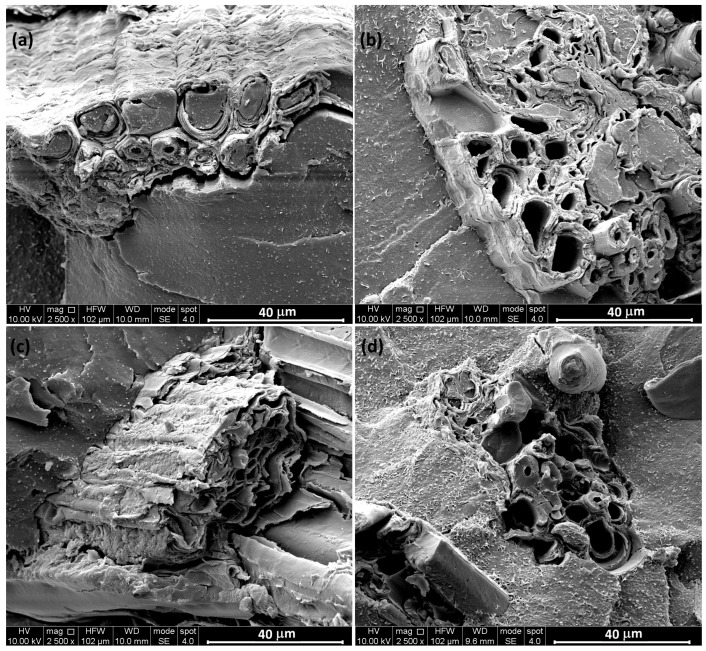
SEM micrographs of biocomposites reinforced with culm-derived particles at 10% (**a**) and 20% (**c**), and 10% (**b**), 20% (**d**) leaf-derived particles.

The morphology exhibited by biocomposites is reported in Figure 2 and Figure 3, where the surface of nitrogen-fractured samples is observed at different magnifications. The SEM micrographs presented in Figure 3 provide compelling evidence that in every analyzed sample, the polymeric matrix successfully infiltrated and filled at least a portion of the fiber lumens. This suggests effective impregnation of the reinforcing fibers by the polymer during the composite fabrication process, which is critical for ensuring good interfacial adhesion and mechanical performance.

**Figure 4:**
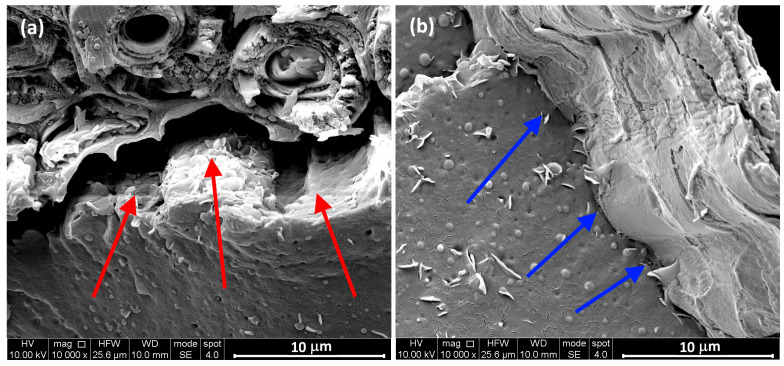
SEM micrographs at higher magnification of biocomposites reinforced with (**a**) culm-derived and (**b**) leaf-derived particles at 10% loading.

Moreover, a closer view of the fractured surface of 10 wt.% loaded biocomposites (Figure 4) provides a detailed visualization of the filler–matrix interface. It is worth noting that the leaf-derived particles show an enhanced interfacial adhesion to the polymer matrix in comparison to their culm-derived counterparts. Indeed, an evident gap between the culm-particle surface and the surrounding matrix (highlighted by the red arrows) can be observed in Figure 4a, indicating weak fiber-matrix adhesion. Conversely, Figure 4b clearly shows the presence of a layer of matrix adhering to the leaf-particle surface (highlighted by the blue arrows) even after the composite failure.

The difference in compatibility of leaf and culm-derived particles with the surrounding polymeric matrix can be ascribed to several aspects, mainly due to variations in their structure and physical properties. Indeed, although the chemical composition is similar between leaves and culm (cellulose, hemicellulose, and lignin), leaf fibers have a more compact structure and higher density (1.139 g/cm^3^ vs. 1.019 g/cm^3^), which promote more effective contact and a larger interfacial area with the polymer matrix. Furthermore, the lower presence of impurities on the surface of leaf-derived particles helps to promote a stronger interface between the matrix and the fillers, leading to good adhesion [48,49]. Additionally, culm fibers absorb more water and contain a higher amount of ash, factors that can hinder adhesion and compatibility with polymers. Finally, the superior mechanical properties of leaf fibers, such as higher strength and elastic modulus, further contribute to enhanced performance in composites, making them more suitable for use as reinforcement in sustainable polymeric materials.

Table 1 reports the theoretical and experimental densities of all biocomposites, along with their corresponding void contents. It is worth noting that the theoretical density values do not agree with the experimental ones due to the presence of voids in the material.

Voids, common manufacturing defects in composites, mainly result from air entrapment during production and moisture absorption during storage. Higher void content increases water penetration susceptibility and leads to reduced and more variable strength properties. In particular, while a good composite should have less than 1% voids, a poorly made one can have up to 5% void content [50].

It is worth noting that void content increases with increasing filler content, irrespective of filler type. This phenomenon may be attributed to the greater entrapment of air in biocomposites during their manufacture as filler content rises. Furthermore, regardless of the filler amount, biocomposites containing leaf-derived particles exhibit lower void content than those with culm-derived particles, which contain more than 5% voids.

This confirms the main findings of the morphological analysis concerning the better morphology of the leaf-reinforced biocomposites, which also explains their enhanced rheological and mechanical behavior in comparison to their culm-reinforced counterparts, as will be detailed in the next paragraphs

### 3.2. Rheological Behavior

**Figure 5:**
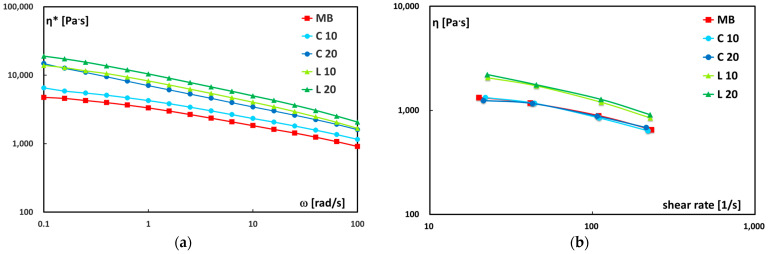
(**a**) Complex viscosity (η*) vs. frequency (ω) and (**b**) shear viscosity (η*) vs. shear rate ($\dot{\gamma }$) for neat Mater-Bi and its biocomposites.

The rheological properties of the neat matrix and biocomposites were investigated to evaluate the effect of lemongrass particles on the viscoelastic behavior. Figure 5a, shows the complex viscosity (η*) as a function of angular frequency (ω). A marked increase in complex viscosity is observed with increasing filler content, particularly for the leaf-based composites, which exhibit higher η* values at both 10 and 20 wt.%. At lower angular frequencies, the presence of solid particles exerts a strong influence on the rheological response, due to the restriction of polymer chain mobility within the filler network. However, as the angular frequency increases, the distinction between the curves becomes less pronounced, indicating that the viscoelastic response tends to be increasingly influenced by the polymer matrix, although the presence of dispersed particles still exerts a non-negligible effect. The observed increase in viscosity with filler content can be attributed to the confinement of the polymer chains between rigid particles, which hinders molecular mobility and enhances resistance to flow. Furthermore, the higher complex viscosity observed in the biocomposites containing leaf particles may result from both improved dispersion of the filler within the matrix and enhanced interfacial compatibility between the two phases, as previously evidenced by SEM analysis. It is important to note that the viscosity increase with the addition of up to 20 wt.% filler remained below one order of magnitude. This relatively modest increase, which contrasts with similar systems using fine particles reported in the literature, can be attributed to the coarse nature of the lemongrass particles. Due to their large size, these particles hinder the formation of an effective three-dimensional network at this specific concentration, suggesting that the system operates below the critical packing fraction or percolation threshold required to induce a drastic viscosity jump.

By examining the steady-shear flow curves obtained with the capillary viscometer (Figure 5b), it can be observed that, at the high shear rates typical of this analysis, the rheological behavior of the studied materials is influenced more by the type of filler than by its content. Composites containing leaf-derived particles display slightly higher viscosity compared to those with culm-derived particles, whose viscosity remains close to that of neat Mater-Bi. These results are consistent with the general trends observed in the parallel plate rheometer, although the influence of the filler appears less pronounced under steady-shear conditions. This minor difference is primarily explained by the distinct deformation modes involved: oscillatory tests (rotational rheometry) probe the structural integrity and elastic network at low strains, while continuous, high-rate shear (capillary rheometry) promotes the partial breakdown or alignment of weak filler–matrix structures. Furthermore, the two techniques explore different shear rate ranges, with capillary rheometry providing data crucial for characterizing the material under typical high-shear industrial processing conditions.

**Figure 6:**
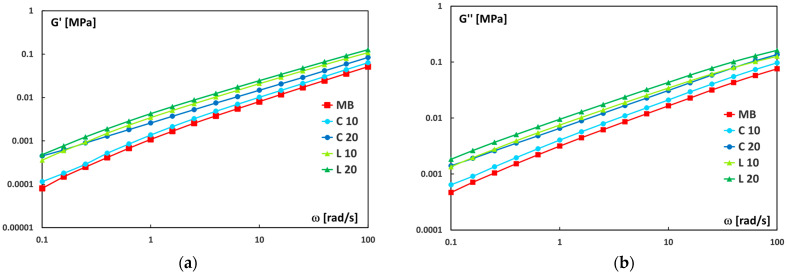
(**a**) Storage modulus (G′) and (**b**) loss modulus (G″) vs. frequency (ω) for neat Mater-Bi and its biocomposites.

The analysis of the storage modulus (G′) and loss modulus (G″) (Figure 6) further supports these findings.

As shown in Figure 6a, G′ increases steadily with angular frequency, indicating a transition toward a more elastic-dominated response at higher frequencies. Likewise, Figure 6b shows a corresponding increase in G″, reflecting greater viscous energy dissipation. These results confirm the viscoelastic nature of the studied systems.

Both moduli (G′ and G″) are higher in the biocomposites than in the neat matrix, with the most pronounced enhancement observed for samples containing leaf-derived particles. This behavior suggests that the presence of leaf particles improves stress transfer between the filler and the polymer matrix, reinforcing both the elastic and viscous components of the material. The observed increase can be attributed to improved interfacial adhesion and more efficient load transfer between the two phases.

### 3.3. Quasi-Static Mechanical Properties

**Figure 7:**
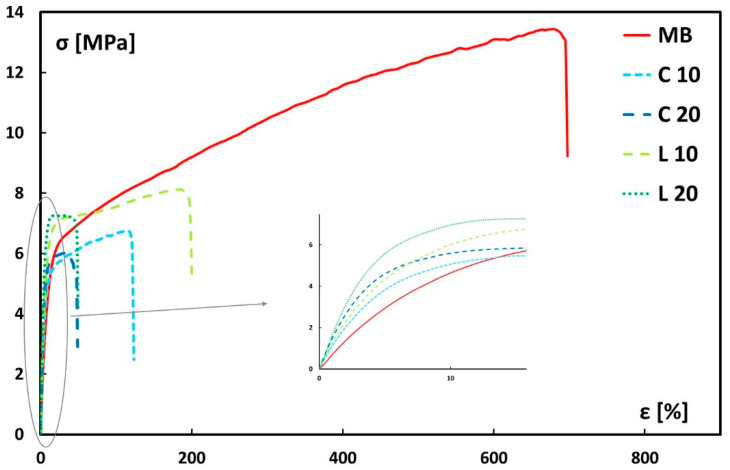
Representative tensile stress–strain curves for neat Mater-Bi and its biocomposites.

Representative tensile stress–strain curves for neat Mater-Bi and its biocomposites are shown in Figure 7.

First of all, it is worth noting that the addition of lemongrass particles dramatically reduces the matrix’s elongation at break, and this effect is particularly evident in systems containing 20 wt.% filler. It is also possible to notice that the tensile strength is lower compared to neat Mater-Bi, and this is attributed to the premature failure of the biocomposite samples.

The data in Table 2 clearly show that the elastic modulus improves as the filler content in the biopolymer matrix increases, a trend evident in both the culm- and leaf-reinforced biocomposites. The observed increase in stiffness is attributed to the filler reinforcing the matrix, as both the leaf- and culm-derived particles have higher stiffness than the neat polymer. However, this effect is notably more pronounced in biocomposites containing particles derived from the leaf. Specifically, the addition of 20 wt.% of leaf-derived particles enables an increase in the tensile modulus of the neat Mater-Bi by about three times (i.e., from 62.6 MPa to 178.9 MPa). On the other hand, at the same amount, the elastic modulus of biocomposites reinforced with culm-derived particles (i.e., 140 MPa) is more than twice that of the neat matrix.

As already stated, this difference can be linked to the distinct morphological and chemical characteristics of the leaf particles, which enhance the interaction with the polymer matrix, leading to greater rigidity compared to particles sourced from the culm. As the filler content increases, the elastic modulus of the composites rises correspondingly, demonstrating the filler’s role in restricting polymer chain mobility. Indeed, the values of the elastic modulus for leaf-reinforced biocomposites at both 10% and 20 wt.% are higher than their culm-based counterparts. This is attributed to the better interfacial adhesion shown by leaf particles with the matrix, as observed in the morphological analysis, as well as their superior mechanical properties [27].

As previously observed from the stress–strain curves, the elongation at break significantly decreases with the addition of filler. Specifically, as the filler content increases, the elongation values progressively decline. Furthermore, it can be noted that biocomposites containing particles derived from the lemongrass leaf exhibit higher deformability compared to those containing particles derived from the culm.

It is worth noting that the tensile strength values of the biocomposites reinforced with 10% lemongrass particles (both leaf-derived and culm-derived particles) are higher than that (i.e., ~6 MPa) of particle board bonded with urea-formaldehyde resin at the same particle content [34]. On the other hand, the latter composites proved to be stiffer than those manufactured using Mater-Bi as the matrix, mainly due to the stiffness mismatch between the polymers. In terms of ductility, regardless of the lemongrass particle type, these biocomposites exhibited significantly higher strain-at-break values than similar biocomposite systems [39,42,47].

Furthermore, the impact of lemongrass particles on the tensile modulus of Mater-Bi based composites is greater than that of other lignocellulosic fillers like grape pomace [37] or brewer’s spent grain [39]. For instance, grape pomace led to basically no modulus improvement at the same 20% weight amount, while brewer’s spent grain, after thermomechanical and chemical treatments, only achieved a doubling of the neat matrix’s modulus at a higher loading of 30 wt.%.

**Figure 8:**
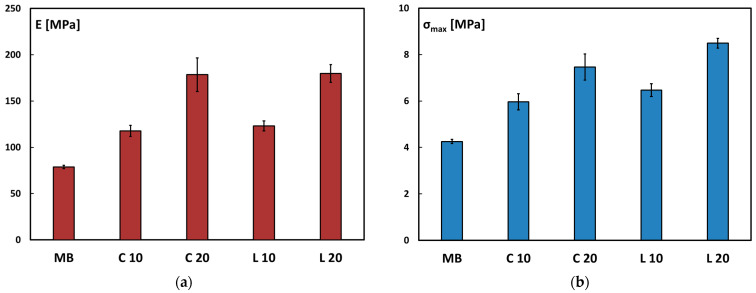
(**a**) Flexural modulus and (**b**) maximum stress for neat Mater-Bi and its biocomposites.

Regarding the flexural behavior of the compared biocomposites, since no specimens failed during the three-point bending test, the discussion will focus on the maximum flexural stress and flexural modulus. The mean values and standard deviations of these flexural properties are reported in the histograms shown in Figure 8a,b.

The flexural modulus of the biocomposites containing equal amounts of culm- and leaf-derived particles exhibit comparable results. This indicates that, under three-point bending loadings, the filler content has a more pronounced influence on the biocomposite’s stiffness than the type of filler kind. Notably, biocomposites with a 20 wt.% filler loading displayed about twice the flexural modulus values than the neat matrix, regardless of the filler type.

The maximum flexural stress values recorded were higher for the biocomposites containing 20 wt.% filler. Overall, when comparing the flexural strength values of the neat matrix, which were lower than those of the biocomposites, it is evident that both types of fillers exerted a reinforcing effect on the Mater-Bi matrix, improving its mechanical strength.

A comparison with the results from the tensile test reveals that the material exhibits different behavior under different loading conditions, while maintaining a coherent trend: as the filler content increases, the tensile test results show a rise in stiffness accompanied by increased brittleness. In contrast, under flexural loading, the increased stiffness is not associated with a loss in ductility, as the specimens remained significantly deformable. This difference is likely attributable to the fact that, under bending, crack propagation is more hindered than in tensile loading conditions.

For the sake of comparison with similar bio-based materials, it is possible to note that the biocomposites manufactured in this work show quite similar flexural strength values but lower flexural modulus values in comparison with Mater-Bi composites reinforced with 30 wt.% brewers’ spent grain [39]. The higher flexural stiffness shown by Mater-Bi/brewers’ spent grain composites can be ascribed to the higher filler content and the modification of the brewers’ spent grain, which involved a thermomechanical process (in a twin-screw extruder) and a chemical treatment (with isophorone diisocyanate).

### 3.4. Dynamic-Mechanical Properties

**Figure 9:**
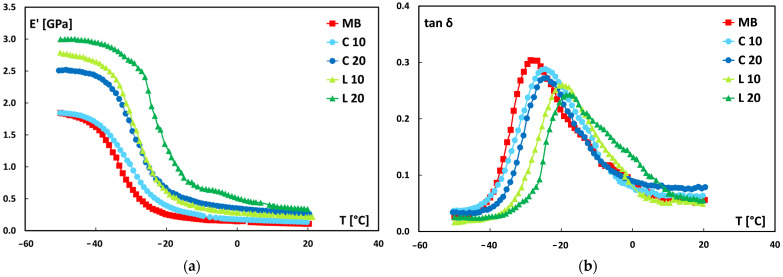
(**a**) Storage modulus and (**b**) damping factor as function of temperature.

The storage modulus (E′) and damping factor (tanδ) trends with temperature for all samples were obtained from dynamic mechanical characterization, as shown in Figure 9.

The storage modulus curves (Figure 9a) provide further evidence of the reinforcing effect of lemongrass particles. At −50 °C, within the fully glassy state where the storage modulus typically exhibits a plateau, all biocomposites display higher E′ values than neat Mater-Bi. This effect is particularly evident for leaf-derived composites, thus confirming the better efficiency of these particles in restricting polymer chain mobility. In addition, the temperature at which the sharp drop in E′ occurs, associated with the glass transition, progressively shifts toward higher values following the same trend, i.e., more pronounced for leaf-derived and higher loading composites. These findings indicate that lemongrass fillers, especially those from leaves, enhance stiffness in the glassy state while simultaneously delaying the onset of the glass transition, thereby improving the overall load-bearing capacity of the biocomposites.

As well known, tanδ provides information about the interfacial adhesion between the matrix and the fiber in composite materials. Weak adhesion results in higher tanδ values, whereas good adhesion reduces the mobility of polymer chains, thereby decreasing damping. A lower tanδ value also indicates that the composite has a good load-bearing capacity.

As shown in Figure 9b, the peaks in the damping factor trend for all the studied biocomposites are shifted to higher temperatures and reduced in height compared with the neat matrix. It suggests that the behavior observed is the result of good interfacial adhesion between the matrix and the fillers incorporated into it, an assumption confirmed by SEM analysis and supported by other studies, which similarly explained the trends observed here.

Specifically, the temperature at which tanδ reaches its maximum is referred to as the glass transition temperature (Tg). This temperature represents the threshold below which the material typically exhibits a glassy behavior, meaning it is rigid and brittle, whereas above this temperature, the material displays ductile characteristics. As observed in the graphs presented in Figure 9b, all the Tg values are well below ambient temperature.

In more detail, whereas the neat matrix shows a Tg of about −28 °C, the biocomposites exhibited increased Tg with rising the filler content. This effect was more pronounced for those reinforced by leaf-derived particles. Indeed, at 20 wt.% filler loadings, the glass transition temperature values for the culm-reinforced and leaf-reinforced biocomposites were −24 °C and −18 °C, respectively. This trend can be attributed to the reduced mobility of the polymer chains caused by the presence of lemongrass particles, particularly those originating from the leaf. Furthermore, the rightward shift of the peak further confirms improved adhesion between the matrix and the filler.

### 3.5. Thermal Behavior

**Figure 10:**
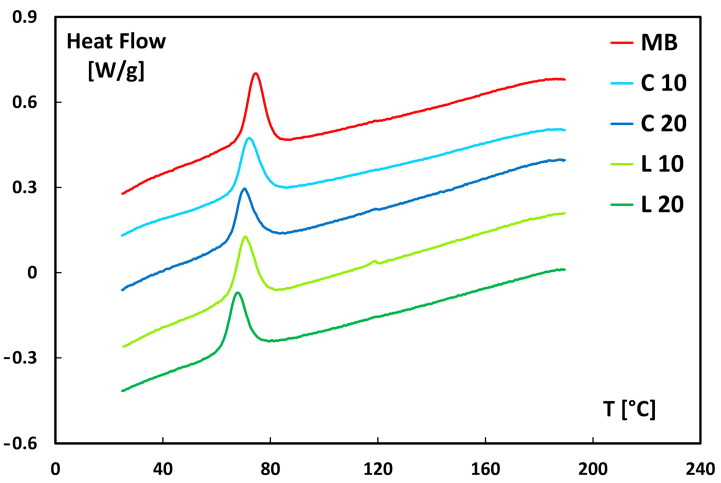
Heat flow thermogram as a function of temperature during the cooling phase.

The calorimetric analysis was conducted to assess the effect of lemongrass particles, sourced from both culm and leaf, on the crystallization ability of the neat polymer matrix. The corresponding cooling thermograms are reported in Figure 10.

The first observation, derived from the thermogram and data reported in Table 3, concerns the reduction in crystallization temperature with increasing filler content within the polymer matrix. This effect is particularly pronounced in biocomposites incorporating leaf-derived particles.

The presence of the filler appears to hinder the crystallization process, likely by interfering with the orderly rearrangement of macromolecular chains, thus requiring a greater degree of undercooling to complete crystallization. This behavior can be attributed to the low nucleating efficiency of the filler within the matrix. Moreover, the area under the crystallization peak of the calorimetric curves for lemongrass particles reinforced biocomposites, directly related to the crystallization enthalpy (ΔH_c_), is lower compared to that of neat Mater-Bi. This finding suggests a reduced crystallization ability of the biocomposites compared to the neat polymer matrix. This effect is likely due to the lignocellulosic particles restricting polymer chain mobility, thereby hindering their ability to align and form an ordered crystalline structure. A similar behavior was found in polymer-based composites reinforced with natural fibers and/or fillers [51].

## 4. Conclusions

The present work successfully developed and characterized biodegradable bio-composites using a Mater-Bi^®^ HF51L2 matrix reinforced with particles derived from lemongrass culm and leaf. Morphological analysis consistently revealed that leaf-derived particles exhibited superior characteristics (i.e., smoother surfaces and fewer impurities), which led to enhanced filler–matrix interfacial adhesion compared to the culm-derived particles.

Rheological investigations showed that both fillers restrict polymer chain mobility and increase the complex viscosity due to filler–matrix interactions. This effect was more pronounced in composites reinforced with leaf-derived particles, reflecting their superior dispersion and compatibility within the matrix.

Mechanical testing revealed that increasing filler content improved the elastic and flexural moduli of the biocomposites. However, leaf-reinforced composites achieved notably higher stiffness values and maintained better deformability than their culm-based counterparts, even though tensile strength and elongation generally decreased due to increased brittleness.

The enhanced filler–matrix interaction in leaf-based composites was further supported by dynamic mechanical thermal analysis. Indeed, these composites exhibited lower tanδ values and higher glass transition temperatures, confirming enhanced load-bearing capacity and reduced polymer chain mobility. Finally, calorimetric studies revealed that the incorporation of lemongrass particles decreases the crystallization temperature and enthalpy of crystallization of the matrix, with a more pronounced effect for leaf-based fillers.

## Figures and Tables

**Table 1:** Theoretical and experimental density and void content of neat Mater-Bi and biocomposites.

Material	Real Density [g/cm^3^]	Theoretical Density [g/cm^3^]	Voids Content [%]
MB	1.260 ± 0.008	N.A.	N.A.
L 10	1.208 ± 0.007	1.246	3.11
L 20	1.180 ± 0.005	1.233	4.33
C 10	1.161 ± 0.009	1.231	5.63
C 20	1.123 ± 0.008	1.203	6.67

**Table 2:** Tensile properties of neat Mater-Bi and its biocomposites.

Material	Tensile Modulus [MPa]	Tensile Strength [MPa]	Strain at Break [%]
MB	62.6 ± 3.8	13.4 ± 0.5	670 ± 80
L 10	113.3 ± 3.4	8.3 ± 0.4	200 ± 60
L 20	178.9 ± 7.6	7.2 ± 0.1	50 ± 20
C 10	95.7 ± 4.7	6.9 ± 0.3	150 ± 40
C 20	140.0 ± 5.3	5.9 ± 0.1	49 ± 15

**Table 3:** Crystallization Enthalpy (ΔH_c_) and crystallization temperature (T_c_) for neat Mater-Bi its and biocomposites.

Material	ΔH_c_ [J/g]	Tc (°C)
MB	−13.3	74.5
L 10	−11.9	70.6
L 20	−10.9	67.5
C 10	−12.0	72.1
C 20	−12.1	70.4

## Data Availability

The data presented in this study are available on request from the corresponding author.
